# Colorectal Cancer Survivors Suffering From Sensory Chemotherapy-Induced Peripheral Neuropathy Are Not a Homogenous Group: Secondary Analysis of Patients’ Profiles With Oxaliplatin-Induced Peripheral Neuropathy

**DOI:** 10.3389/fphar.2021.744085

**Published:** 2021-11-04

**Authors:** Nicolas Kerckhove, Marie Selvy, Céline Lambert, Coralie Gonneau, Gabrielle Feydel, Caroline Pétorin, Agnès Vimal-Baguet, Sergey Melnikov, Sharif Kullab, Mohamed Hebbar, Olivier Bouché, Florian Slimano, Vincent Bourgeois, Valérie Lebrun-Ly, Frédéric Thuillier, Thibault Mazard, David Tavan, Kheir Eddine Benmammar, Brigitte Monange, Mohamed Ramdani, Denis Péré-Vergé, Floriane Huet-Penz, Ahmed Bedjaoui, Florent Genty, Cécile Leyronnas, Jérôme Busserolles, Sophie Trévis, Vincent Pinon, Denis Pezet, David Balayssac

**Affiliations:** ^1^ INSERM U1107 NEURO-DOL, Université Clermont Auvergne, Clermont-Ferrand, France; ^2^ Délégation à La Recherche Clinique et à L’Innovation, CHU Clermont-Ferrand, Clermont-Ferrand, France; ^3^ Institut Analgesia, Université Clermont Auvergne, Clermont-Ferrand, France; ^4^ Service Oncologie Digestive, CHU Clermont-Ferrand, Clermont-Ferrand, France; ^5^ Service Chirurgie Générale et Viscérale, Centre Hospitalier de Saint-Flour, Saint-Flour, France; ^6^ Service Oncologie, Centre Hospitalier de Moulins Yzeure, Moulins, France; ^7^ CHRU Lille, Service Oncologie, Lille, France; ^8^ Service Oncologie Digestive, CHU Reims, Université de Reims Champagne-Ardenne, Reims, France; ^9^ Service Pharmacie, CHU Reims, BioSpect, SFR CAP-Santé, Université de Reims Champagne-Ardenne, Reims, France; ^10^ Service Oncologie Digestive, Centre Hospitalier de Boulogne sur Mer, Boulogne-Sur-Mer, France; ^11^ Service Oncologie, CHU Limoges, Limoges, France; ^12^ IRCM, Inserm, Univ Montpellier, ICM, Montpellier, France; ^13^ Service Gastro-entérologie, Infirmerie Protestante de Lyon, Caluire et Cuire, France; ^14^ Service Oncologie, Centre Hospitalier Emile Roux, Le Puy-en-Velay, France; ^15^ Service Gastro-entérologie, Centre Hospitalier de Béziers, Béziers, France; ^16^ Service Hépato-gastro-entérologie, Centre Hospitalier Saint-Joseph Saint-Luc, Lyon, France; ^17^ Service Gastro Entérologie, Centre Hospitalier Alpes Leman, Contamine sur Arve, France; ^18^ Service Gastro-entérologie, Centre Hospitalier Intercommunal Les Hôpitaux Du Léman, Thonon Les Bains, France; ^19^ Service Chirurgie Digestive et Viscérale, Centre Hospitalier de Vichy, Vichy, France; ^20^ Service Oncologie, Groupe Hospitalier Mutualiste de Grenoble, Grenoble, France; ^21^ Service Pharmacie, CHU Clermont-Ferrand, Clermont-Ferrand, Clermont-Ferrand, France; ^22^ INSERM, M2iSH, USC-INRA 2018, Université Clermont Auvergne, CHU Clermont-Ferrand, Clermont-Ferrand, France

**Keywords:** chemotherapy-induced peripheral neuropathy, neuropathic pain, colorectal cancer, oxaliplatin, cluster analysis

## Abstract

Oxaliplatin, a pivotal drug in the management of colorectal cancer, causes chemotherapy-induced peripheral neuropathy (CIPN) in a third of cancer survivors. Based on a previous cross-sectional study assessing oxaliplatin-related sensory CIPN in colorectal cancer survivors, a secondary analysis was designed to explore the possibility that different clusters of patients may co-exist among a cohort of patients with oxaliplatin-related CIPN. Other objectives were to characterize these clusters considering CIPN severity, anxiety, depression, health-related quality of life (HRQOL), patients’ characteristics and oxaliplatin treatments. Among the 96 patients analyzed, three clusters were identified (cluster 1: 52, cluster 2: 34, and cluster 3: 10 patients). Clusters were significantly different according to CIPN severity and the proportion of neuropathic pain (cluster 1: low, cluster 2: intermediate, and cluster 3: high). Anxiety, depressive disorders and HRQOL alteration were lower in cluster 1 in comparison to clusters 2 and 3, but not different between clusters 2 and 3. This study underlines that patients with CIPN are not a homogenous group, and that CIPN severity is associated with psychological distress and a decline of HRQOL. Further studies are needed to explore the relation between clusters and CIPN management.

## 1 Introduction

Oxaliplatin is a key anticancer drug in the management of colorectal cancer. However, oxaliplatin is one of the most neurotoxic anticancer drugs and responsible for chemotherapy-induced peripheral neuropathy (CIPN). More precisely, oxaliplatin induces acute neuronal hyperexcitability, occurring shortly after infusion, mainly characterized by cold hypersensitivity of the distal extremities and of the orofacial area (Gebremedhn et al., 2018). This acute neurotoxicity can affect up to 98% of patients (Gebremedhn et al., 2018). Thereafter, the repetition of the chemotherapy cycles can be responsible for chronic CIPN, typically described as distal and symmetric sensitive disorders, such as dysesthesia/paresthesia and to a lesser extent neuropathic pain, affecting the hands and feet (Alberti, 2019). Oxaliplatin-associated CIPN has persisted for several years after the end of treatment in several cancer survivors (5 years after the end of chemotherapy: 31.3% [95% confidence interval (CI): 26.8; 36.0]), and is associated with psychological distress, a decrease of health-related quality of life (HRQOL) (Selvy et al., 2020) and a greater risk of patients falling, with all the negative consequences that can result from this (Kolb et al., 2016).

Patients with CIPN are frequently described as a homogenous population of patients with symptoms of neuropathy. But recently, Wang et al. identified four symptom clusters among patients with CIPN (i.e. sensory neuropathy symptoms, mixed motor-sensory neuropathy symptoms, mixed sensorimotor neuropathy symptoms, and autonomic neuropathy symptoms) (Wang et al., 2019). This study was the only one to underline that CIPN is predominantly a sensory neuropathy but that some patients may present mixed motor-related and autonomic symptoms. Moreover, neuropathic pain is frequently associated with CIPN symptomatology, whereas less than half of patients present pain symptoms (de Carvalho Barbosa et al., 2014; Selvy et al., 2020).

Cluster analysis is a statistical technique that aims to identify homogenous groups of patients characterized by their responses to a set of variables. Cluster analysis can be used to complement the problem-oriented approach by assessing the existence and size of patient groups with systematically poorer experiences across a set of variables. These patient groups can then be profiled by describing intra-group characteristics, and quality problems within groups might be explored to better target quality improvement initiatives (Bjertnaes et al., 2013). Knowledge of such subgroups of patients is valuable for tailoring and implementing management quality (Bjertnaes et al., 2013).

Based on a previous cross-sectional study assessing oxaliplatin-related sensory CIPN in colorectal cancer survivors (Selvy et al., 2020), the main objective of this secondary analysis was to explore the possibility that different clusters of patients may co-exist among a cohort of patients with oxaliplatin-related CIPN. The secondary objective was to characterize these clusters of patients considering CIPN severity and symptoms, anxiety, depression, HRQOL, patients’ characteristics and oxaliplatin treatments.

## 2 Materials and Methods

### 2.1 Study Design

This secondary analysis is based on a previous multicenter cross-sectional study assessing the prevalence and severity of CIPN in survivors of colorectal cancer, 5 years after the end of oxaliplatin-based chemotherapy (Selvy et al., 2020). This previous study also assessed the prevalence of neuropathic pain, anxiety, depression, and HRQOL. Patients were assessed once using a self-administered questionnaire, and no longitudinal assessment was performed (Selvy et al., 2020).

In the present cluster analysis, the main objective was to explore the existence of different clusters (subgroups) of patients among patients with oxaliplatin-related CIPN. The secondary objectives were to characterize these clusters according to anxiety, depression, HRQOL, patients’ characteristics and oxaliplatin treatments.

The study conformed to the Strengthening the Reporting of Observational Studies in Epidemiology (STROBE) guidelines (von Elm et al., 2007), and the protocol was registered on ClinicalTrials.gov (NCT02970526). The study was approved by a local ethics committee (Comité de Protection des Personnes sud-est 6, IRB: 00008526, No. 2016/CE16, 26/02/2016) and carried out anonymously. It was approved by the Advisory Committee on the Treatment of Research Information (No. 15.645, 13/05/2015). Consent was obtained from all the participants by telephone.

### 2.2 Setting

The study was coordinated by the University Hospital of Clermont-Ferrand (CHU Clermont-Ferrand, France). The patients were recruited from 16 French centers (University Hospitals: CHU Clermont-Ferrand, CHU Limoges, CHU Reims, CHRU Lille, and Institut du Cancer Montpellier; General Hospitals: CH Saint-Flour, CH Moulins, CH Boulogne-sur-Mer, CH Béziers, CH Puy en Velay, Infirmerie Protestante de Lyon, CH Saint-Joseph Saint Luc Lyon, CH Alpes Leman, CHI Les Hôpitaux du Léman, CH Vichy, and GHM Grenoble) from 21 June 2016 until 29 August 2019.

### 2.3 Participants

The inclusion criteria have been already described (Selvy et al., 2020), and for this secondary analysis were as follows: sensory scores of the QLQ-CIPN20 ≥ 30/100, treatment with adjuvant oxaliplatin-based chemotherapy (FOLFOX-4) for colorectal cancer, ≤5 years from the time chemotherapy was discontinued, and no cancer relapse during these 5 years (cancer survivors). The exclusion criteria were sensory scores of the QLQ-CIPN20 < 30/100, age <18 years, and patients with neurological diseases (stroke, Parkinson’s disease, Alzheimer’s disease). Missing data on any of the variables were added to the exclusion criteria.

Patients were identified from the database of the chemotherapy prescription software of each participating center. Thereafter, according to the inclusion/exclusion criteria, each center phoned their patients to inquire whether they would participate in the study. After patient acceptance, a paper questionnaire and a stamped envelope for the response were sent to the patient. Patients returned their questionnaires to the coordinating center, where their responses were recorded and analyzed (For more details see Selvy et al. (Selvy et al., 2020)).

### 2.4 Variables

The primary endpoint was the sensory score of the EORTC QLQ-CIPN20 (Lavoie Smith et al., 2013), which rates CIPN severity from 0 (least) to 100 (worst) during the last week (Postma et al., 2005) (for scoring see: https://www.eortc.org/app/uploads/sites/2/2018/02/SCmanual.pdf). Sensory CIPN was defined as a sensory QLQ-CIPN20 score of ≥30/100 in the present study, based on the work by Alberti et al. (2014) (Alberti et al., 2014).

With regards to the secondary endpoints, ongoing neuropathic pain was defined as a visual analogue scale (VAS) score ≥40/100 and a DN4 (French abbreviation: Douleur Neuropathique *4*, for neuropathic pain 4) interview questionnaire score ≥3/7 (Bouhassira et al., 2005). Anxiety and depression were assessed using the Hospital Anxiety and Depression Scale (HADS) questionnaire at the time of the answer (normal: ≤7/21, borderline or suggestive of anxiety/depression: 8–10/21, indicative of anxiety/depression: ≥11/21) (Zigmond and Snaith, 1983). The patients’ HRQOL at the time of the answer was assessed using the EORTC QLQ-C30 (global health status, functional scales, physical functioning, role functioning, emotional functioning, cognitive functioning, social functioning, fatigue, nausea and vomiting, pain, dyspnea, insomnia, appetite loss, constipation, diarrhea, financial difficulties) (Aaronson et al., 1993). The patients’ oncological treatment characteristics, including cumulative dose (mg/m^2^), dose intensity (mg/m^2^/week), and the dates of the first and last oxaliplatin cycles were recorded. Socio-demographiccharacteristics were recorded, including gender, age, daily cigarette use, and hazardous alcohol use (males: ≥21 alcohol units/week and females: ≥14 alcohol units/week), at the time of the answer. Weight variation between the first and the last chemotherapy cycle, and body mass index (BMI) at the first chemotherapy cycle were recorded.

### 2.5 Data Sources and Measurements

Data assessing CIPN, neuropathic pain, anxiety, depression, and HRQOL were obtained from the completed questionnaire. Oncological data and patients’ characteristics were obtained from the chemotherapy prescription software of each center. All the data were recorded and managed using REDCap electronic data capture tools hosted at CHU Clermont-Ferrand (Harris et al., 2009).

### 2.6 Data Selection

Patients’ (gender, age, tobacco, hazardous alcohol, BMI first cycle, weight variation) and treatment characteristics (cumulative dose, dose intensity, time since last administration) were collected to characterize and compare our patients. Several studies have shown that weight and BMI (Aprile et al., 2008; Shahriari-Ahmadi et al., 2015), gender and age (Vincenzi et al., 2013; Molassiotis et al., 2019; Selvy et al., 2020), tobacco and alcohol (Kawakami et al., 2012; Scott et al., 2013; Vincenzi et al., 2013; Molassiotis et al., 2019), and chemotherapy regimen (Beijers et al., 2014; Molassiotis et al., 2019; Selvy et al., 2020) are related to chemotherapy toxicity in colorectal cancer patients. Items of QLQ-CIPN20 were collected to characterize (sensory, motor and autonomic symptoms) and determine the intensity of CIPN. The QLQ-CIPN20 is a validated questionnaire of EORTC (Lavoie Smith et al., 2013) that provided detailed information, distinguished more subtle degrees of neuropathy, and was more responsive to change over time than the NCI-CTCAE (Le-Rademacher et al., 2017). Data on neuropathic pain (VAS ≥4/10 + DN4 interview ≥3/7), a common symptom affecting about 30% of patients with CIPN and considered as a sign of aggravation (de Carvalho Barbosa et al., 2014; Selvy et al., 2020), was collected to determine the presence of pain with a neuropathic component or not. Items of QLQ-C30 (questionnaire of EORTC) were collected to determine the HRQOL of patients which is degraded in the majority of patients with CIPN (Selvy et al., 2020; Bonhof et al., 2021). Items of HADS were collected to highlight the presence of anxiety and depressive disorders, which are well-known to be comorbidities associated with CIPN and neuropathic pain (Bonhof et al., 2019; Selvy et al., 2020).

### 2.7 Statistical Methods

Statistical analyses were performed using Stata software (version 15, StataCorp, College Station, US) and R 3.5.1 (http://cran.r-project.org/). All the tests were two-sided with a type I error set at 0.05. Categorical variables were expressed as number of patients and percentages, and quantitative variables as mean ± standard deviation or as median [interquartile range], according to statistical distribution. Normality was verified by the Shapiro-Wilk test and/or histogram. A multiple correspondence analysis (MCA), which can be considered as a generalization of principal component analysis for categorical rather than quantitative variables, was applied to study the associations between the sociodemographic (e.g. age, gender, BMI) and chemotherapy (e.g. cumulative dose, dose intensity) characteristics of the patients, QLQ-CIPN20, neuropathic pain, anxiety, depression and HRQOL. For this analysis, variables were chosen according to clinical relevance and statistical distribution (characteristics always present or always absent were not considered). Quantitative variables were transformed into qualitative ones according to pre-existing validated categories if applicable or considering terciles to obtain similar categories in terms of numbers of patients. Only individuals without missing data were used for the MCA, and they were compared to the excluded ones in order to study the representativeness of the sample (with chi-squared test, Student’s t test or Mann-Whitney test). Then, a mixed unsupervised classification (k-means clustering applied to the partition obtained from an ascending hierarchical classification using Ward’s distance) was used to determine groups of patients. Finally, the three clusters obtained were compared with the chi-squared test or Fisher’s exact test for qualitative variables (followed by the Marascuilo procedure if the omnibus *p*-value was less than 0.05), and with ANOVA or the Kruskal-Wallis test for quantitative ones, followed by the Tukey-Kramer test and Dunn’s test, respectively, if appropriate.

## 3 Results

**FIGURE 1:**
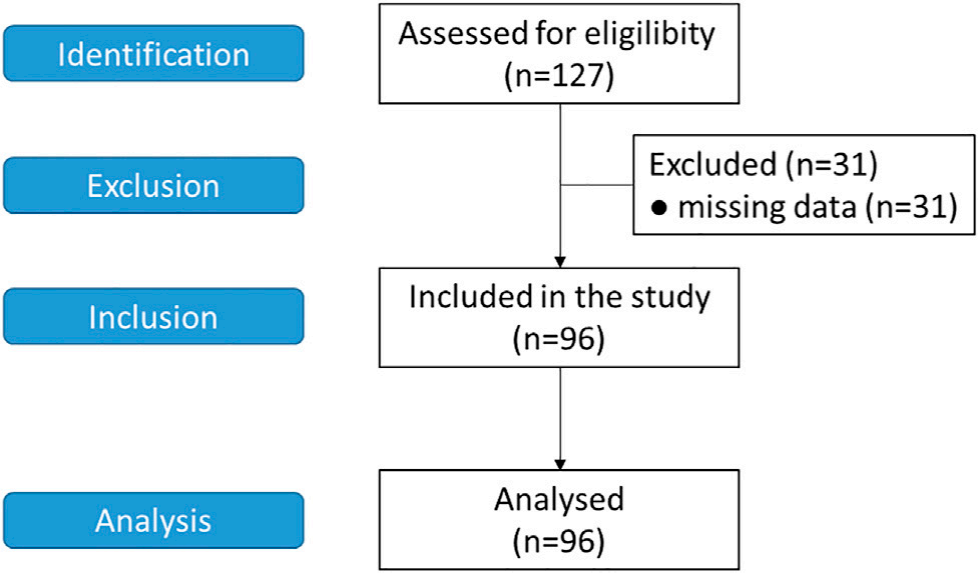
Study flowchart.

Among the initial 127 patients with an oxaliplatin-related CIPN (Selvy et al., 2020), 96 patients were included and 31 excluded because of data missing from any of the variables selected for the analysis (Figure 1). Excluded patients had higher sensory CIPN scores than included patients (54.1 ± 16.5 vs 46.8 ± 13.3, *p* = 0.01). The characteristics of the selected patients are presented in Table 1. Among the 96 patients analyzed, three clusters (cluster 1: 52 patients, cluster 2: 34 patients, and cluster 3: 10 patients) were identified (Figure_supplementary file).

**TABLE 1:** Characteristics of the patients analyzed. Data are presented as number (percentages), mean ± standard deviation or median [interquartile range]. Hazardous alcohol use (males: ≥21 alcohol units/week and females: ≥14 alcohol units/week). BMI: body mass index.

Items	Total (*n* = 96)
Female	46 (47.9)
Age (years)	67.6 ± 7.7
<65	31 (32.3)
65–70	32 (33.3)
>70	33 (34.4)
Weight variation (%)	0.0 [-3.4; 3.3]
Decrease	31 (32.3)
Stable	33 (34.4)
Increase	32 (33.3)
BMI (kg/m^2^) 1st cycle	25.3 ± 5.1
<25	54 (56.2)
25–30	23 (24.0)
≥30	19 (19.8)
Tobacco (daily)	13 (13.5)
Alcohol (hazardous use)	11 (11.5)
Oxaliplatin treatment Time since last infusion (years)	1.9 [1.0; 3.3]
<1 year	24 (25.0)
[1, 2 [ years	27 (28.1)
[2, 3 [ years	18 (18.8)
[3, 4 [ years	13 (13.5)
≥4 years	14 (14.6)
Cumulative dose (mg/m^2^)	1,268 ± 437
<1,000	26 (27.1)
1,000–1,500	36 (37.5)
>1,500	34 (35.4)
Dose intensity (mg/m^2^/day)	9.4 ± 2.8
<10	56 (58.3)
≥10	40 (41.7)

**FIGURE 2:**
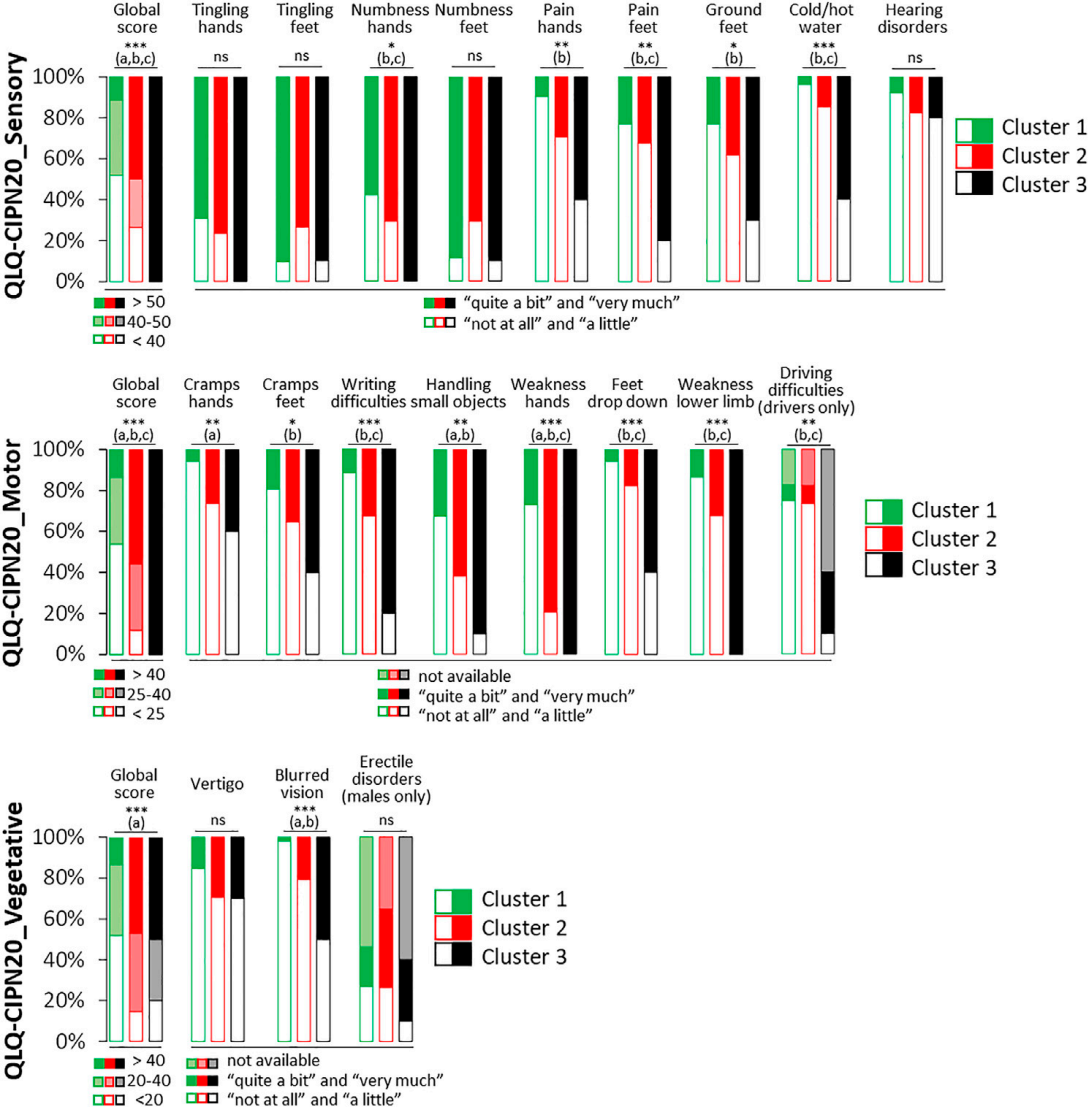
Scores of the QLQ-CIPN20 (sensory, motor and vegetative) among the three clusters of patients with a sensory CIPN. Omnibus *p*-value: *p < 0.05; **p < 0.01; ***p < 0.001; ns, not significant. Significant difference (*p* < 0.05, post hoc analysis) between: a, cluster 1 and cluster 2; b, cluster 1 and cluster 3; c: cluster 2 and cluster 3.

Regarding the demographic and chemotherapy characteristics of these three clusters of patients (Table 2), no difference was observed, except for BMI, for which cluster 1 had a lower BMI than clusters 2 and 3. Concerning neuropathic symptoms, cluster 3 had higher global QLQ-CIPN20 sensory scores than the other clusters, while those of cluster 2 were higher than those of cluster 1. Moreover, the clusters can be distinguished from each other if we look more precisely at the sensory, motor and vegetative symptoms (Figure 2). Cluster 1 could be considered as patients with slight neuropathic symptoms, cluster 3 as patients with severe neuropathic symptoms, and cluster 2 as an intermediate subgroup. Interestingly, cluster 3 is distinguished from the other clusters by the more frequent presence of numbness in the hands, pain in the feet, disorders of sensitivity to heat/cold, disorders of fine motor skills (difficulty in writing), weakness in the feet/hands, and foot drop. Note that few differences between clusters were found concerning vegetative disorders, except for blurred vision which was scarcely present in cluster 1 in comparison to the other clusters.

**TABLE 2:** Characteristics of the patients according to their cluster. Data are presented as number (percentages), mean ± standard deviation or median [interquartile range]. Hazardous alcohol use (males: ≥21 alcohol units/week and females: ≥14 alcohol units/week). Weight variations were defined as follows: decrease (-2% between first oxaliplatin infusion and last one), increase (+2%) and stable (less than -2 and 2%). BMI: body mass index. Significant difference (*p* < 0.05, post hoc analysis) between: a, cluster 1 and cluster 2; b, cluster 1 and cluster 3; c: cluster 2 and cluster 3.

Items	Cluster 1 *n* = 52	Cluster 2 *n* = 34	Cluster 3 *n* = 10	*p*-values
Female	28 (53.8)	12 (35.3)	6 (60.0)	0.17
Age (years)	68.3 ± 7.8	66.8 ± 8.4	66.7 ± 4.9	0.61
<65	15 (28.9)	11 (32.4)	5 (50.0)	0.48
65–70	18 (34.6)	13 (38.2)	1 (10.0)	
>70	19 (36.5)	10 (29.4)	4 (40.0)	
Weight variation (%)	0.0 [-4.1; 2.5]	0.9 [-2.5; 3.9]	0.0 [-1.8; 1.7]	0.26
Decrease	20 (38.5)	9 (26.5)	2 (20.0)	0.17
Stable	18 (34.6)	9 (26.5)	6 (60.0)	
Increase	14 (26.9)	16 (47.1)	2 (20.0)	
BMI (kg/m^2^) 1st cycle	24.1 ± 4.3	26.8 ± 5.8	26.7 ± 5.3	0.03^a^
<25	34 (65.4)	16 (47.1)	4 (40.0)	0.03^b^
25–30	13 (25.0)	9 (26.5)	1 (10.0)	
≥30	5 (9.6)	9 (26.5)	5 (50.0)	
Tobacco (daily)	8 (15.4)	5 (14.7)	0 (0.0)	0.57
Alcohol (hazardous use)	5 (9.6)	4 (11.8)	2 (20.0)	0.52
Oxaliplatin treatment Time since last infusion (years)	2.0 [1.2; 3.3]	2.0 [0.8; 3.3]	1.0 [0.5; 2.8]	0.43
<1 year	9 (17.3)	10 (29.4)	5 (50.0)	0.33
[1, 2 [ years	18 (34.6)	7 (20.6)	2 (20.0)	
[2, 3 [ years	9 (17.3)	8 (23.5)	1 (10.0)	
[3, 4 [ years	7 (13.5)	6 (17.7)	0 (0.0)	
≥4 years	9 (17.3)	3 (8.8)	2 (20.0)	
Cumulative dose (mg/m^2^)	1,231 ± 413	1,353 ± 451	1,175 ± 503	0.30
<1,000	15 (28.9)	8 (23.5)	3 (30.0)	0.53
1,000–1,500	22 (42.3)	10 (29.4)	4 (40.0)	
>1,500	15 (28.9)	16 (47.1)	3 (30.0)	
Dose intensity (mg/m^2^/day)	8.9 ± 2.6	10.1 ± 3.1	9.6 ± 2.6	0.16
<10	35 (67.3)	16 (47.1)	5 (50.0)	0.14
≥10	17 (32.7)	18 (52.9)	5 (50.0)	

**FIGURE 3:**
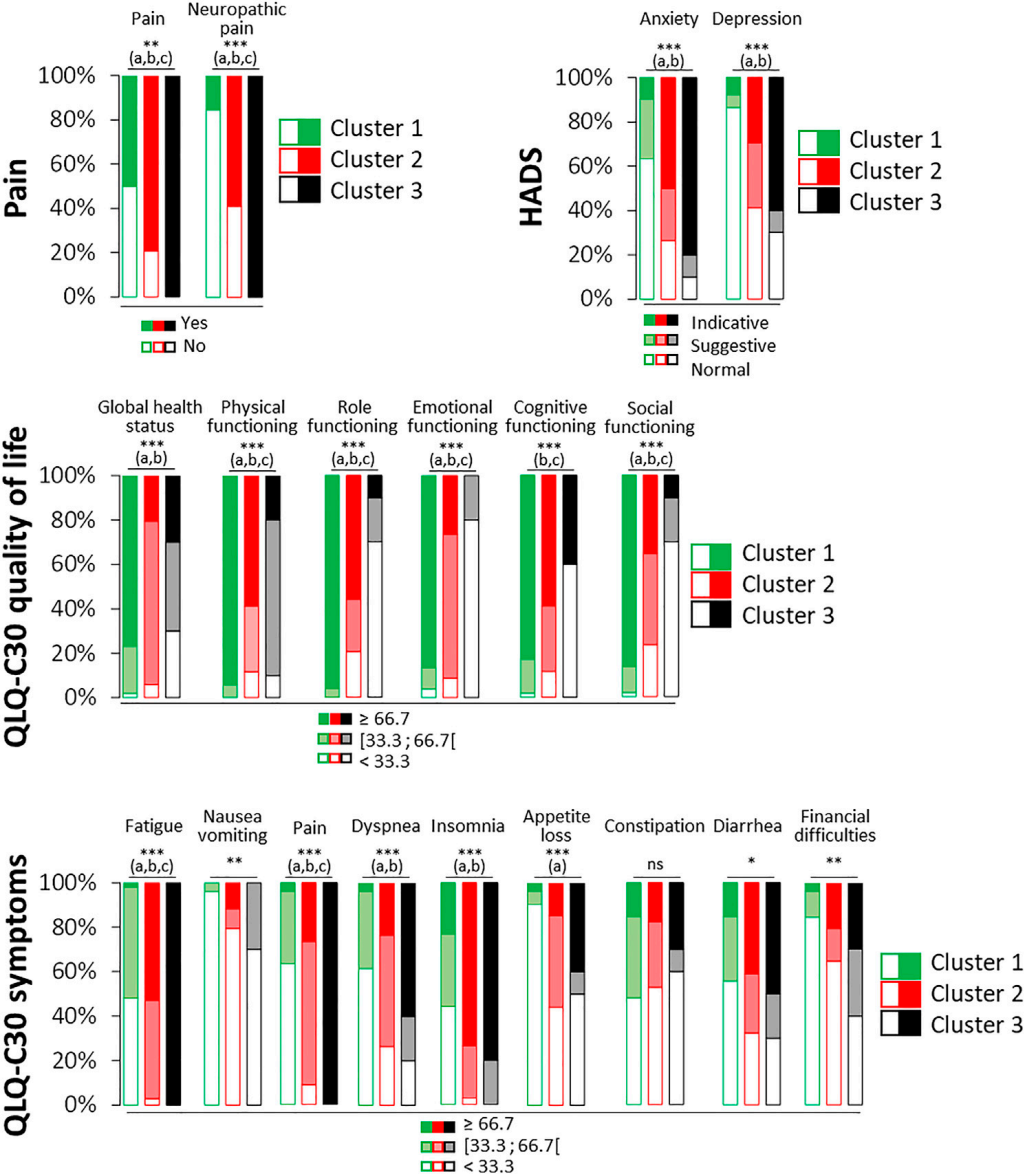
Characteristics of pain, anxiety, depression and HRQOL among the four clusters of patients with a sensory CIPN. Anxiety and depression were assessed thanks to the HADS questionnaire and classified according to normal (≤7/21), suggestive (8–10/21) and indicative (≥11/21) scores of anxiety or depression. HRQOL was assessed thanks to the QLQ-C30 questionnaire and classified according each third of the scores (<33.3 [33.3–66.7 [, and ≥66.7) for the global health scale, the functional scales (physical, role functioning, emotional, cognitive, and social), and the symptomatic scales (fatigue, nausea/vomiting, dyspnea, insomnia, appetite loss, constipation, diarrhea, and financial difficulties). Omnibus *p*-value: *p < 0.05; **p < 0.01; ***p < 0.001; ns, not significant. Significant difference (*p* < 0.05, post hoc analysis) between: a, cluster 1 and cluster 2; b, cluster 1 and cluster 3; c: cluster 2 and cluster 3.

Focusing on the pain symptoms (Figure 3), cluster 1 consists of patients with the least pain and neuropathic pain, and vice versa for cluster 3. Noteworthy, cluster 2 had intermediate proportions of pain and neuropathic pain in comparison to cluster 1 and cluster 3, respectively. Finally, the assessment of psychological distress (Figure 3) highlighted that cluster 1 had less indicative scores of anxiety and more normal scores of depression, better scores of HRQOL (QLQ-C30 quality of life and symptoms) than the other clusters, especially cluster 3 which is its opposite. Cluster 2 is an intermediate group. Role, emotional, cognitive and social functioning scores were significantly lower for cluster 3 in comparison to the other clusters. Conversely, physical, role, emotional and social functioning scores were significantly higher for cluster 1 in comparison to the other clusters. Fatigue scores were significantly different between each cluster. Cluster 1 had the lower scores, cluster 3 the highest ones and cluster 2 intermediate ones. Cluster 1 had also the lowest scores of insomnia in comparison to other clusters.

**FIGURE 4:**
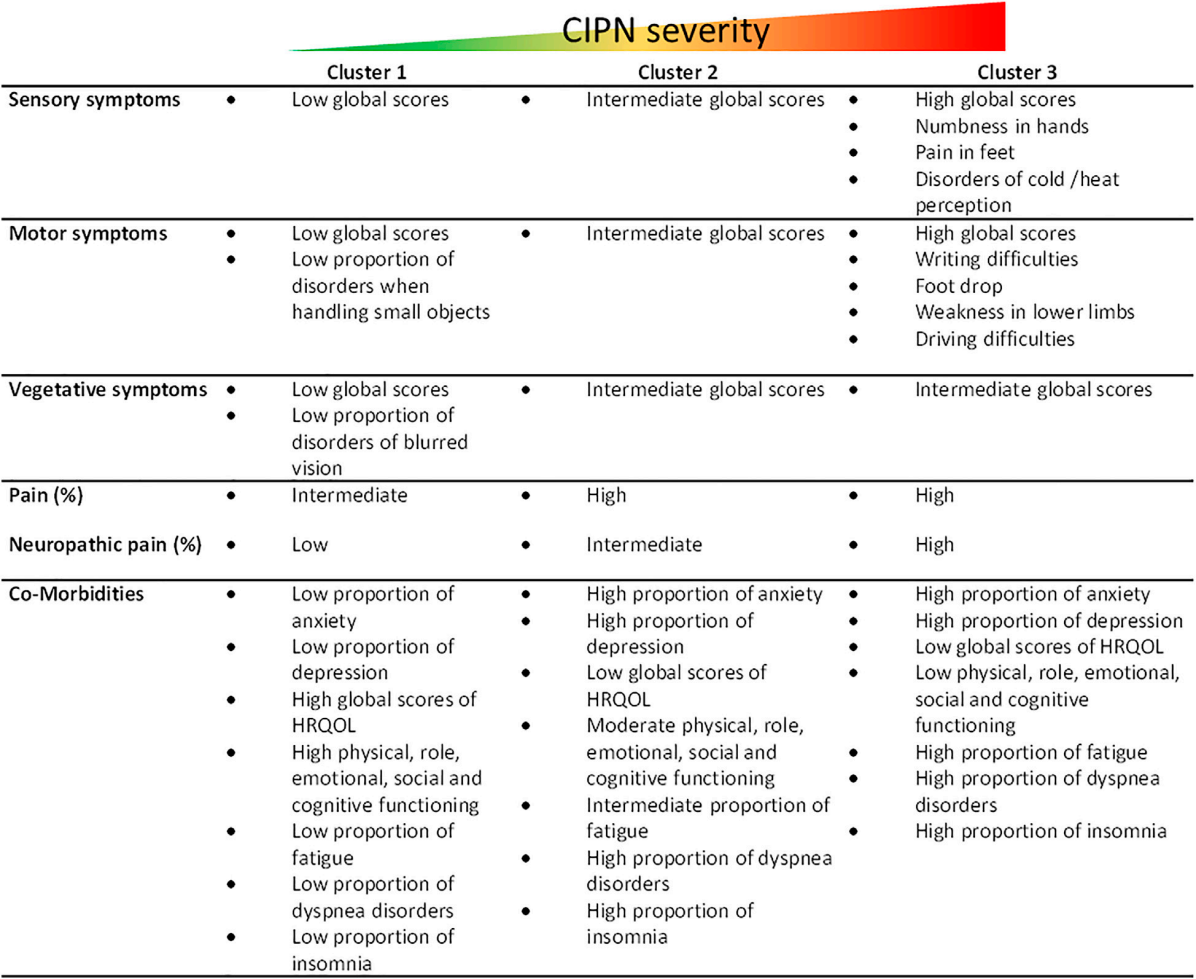
Summary of cluster characteristics. Figure_supplementary file: Multiple correspondence analysis (MCA) plot of variables **(A)** and patients **(B)**. QLQ-CIPN20–17 (3 + 4): QLQ-CIPN20 questionnaire blurred vision, score “quite a bit” and “very much” HADS depression [8; 10]: HADS questionnaire, depression scale, suggestive scores between 8 and 10.QLQ-C30 - AP ≥ 66.7: QLQ-C30 questionnaire appetite loss, scores higher than or equal to 66.7. QLQ-C30 - AP [33.3; 66.7[: QLQ-C30 questionnaire appetite loss, scores between 33.3 and 66.7 (66.7 excluded). QLQ-C30 - EF < 33.3: QLQ-C30 questionnaire emotional functioning, scores lower than 33.3. QLQ-C30-CF < 33.3: QLQ-C30 questionnaire cognitive functioning, scores lower than 33.3. QLQ-C30-CF [33.3; 66.7 [: QLQ-C30 questionnaire cognitive functioning, scores between 33.3 and 66.7 (66.7 excluded). QLQ-C30-FA < 33.3: QLQ-C30 questionnaire fatigue, scores lower than 33.3. QLQ-C30-PA ≥ 66.7: QLQ-C30 questionnaire pain, scores higher than or equal to 66.7. QLQ-C30-RF < 33.3: QLQ-C30 questionnaire role functioning, scores lower than 33.3. QLQ-C30-RF [33.3; 66.7 [: QLQ-C30 questionnaire role functioning, scores between 33.3 and 66.7 (66.7 excluded) QLQ-C30-QOL <33.3: C30 questionnaire global health status, scores lower than 33.3 QLQ-C30-SF < 33.3: QLQ-C30 questionnaire social functioning, scores lower than 33.3. QLQ-C30 - SL < 33.3: QLQ-C30 questionnaire insomnia, scores lower than 33.3.

Overall, we can summarize the different clusters as such in Figure 4, and propose relationships between the presence of certain neuropathic symptoms and the severity of CIPN.

## 4 Discussion

The purpose of this secondary study was to investigate the presence of different subgroups in a population of patients with CIPN, in this case after an adjuvant oxaliplatin-based regimen for colorectal cancer. The identification of patient subgroups and their distinct characteristics would improve knowledge of the pathophysiology of CIPN, as well as their management, including the identification of signals of CIPN worsening. This is all the more important as there is currently no preventive or curative treatment for CIPN (except for duloxetine [only for the symptom “neuropathic pain”]) (Loprinzi et al., 2020) and its diagnosis and management remain far from optimal (Selvy et al., 2021b). Thus, a finer characterization of patients could lead to more personalized and effective management. Previous studies, including two recent ones, have already characterized patients suffering from CIPN, but only regarding their neuropathic symptoms (Wang et al., 2019; Zhi et al., 2021). The interest of our study and its innovative aspect is that it includes sociodemographic factors, HRQOL, and comorbidities in this characterization.

Our study was able to identify three subgroups (clusters) of neuropathic patients. These different clusters are mainly distinguished by the severity of their neuropathic symptoms and their comorbidities, going gradually from cluster 1 (less severe) to cluster 3 (more severe). Conversely, these clusters were not driven by the sociodemographic characteristics of the patients or the chemotherapy regimen, whereas several risk factors have been associated with oxaliplatin-related CIPN, such as younger age (<60 years), body surface area (because of larger dose of oxaliplatin administered), higher body weight, female, cumulative dose, diabetes, and smoking (Pulvers and Marx, 2017; Staff et al., 2019). The fact that we found no impact of some of these risk factors in our study suggests that they are not related to the severity or worsening of oxaliplatin-related neuropathic disorders. Nevertheless, this result must be considered with caution due to the small number of patients studied, which limits the identification of risk factors.

Concerning neuropathic symptoms, a gradation clearly appears with cluster 1 consisting of patients with mild CIPN severity of and associated comorbidities, as opposed to cluster 3. We therefore focused on cluster 3 to identify possible characteristics that could indicate a CIPN severity/worsening. The results obtained show that the most severe patients have neuropathic disorders characterized more by disorders in the lower limbs (pain, motor disorders, muscle weakness, but no numbness in feet), sensory disorders in the distinction of hot and cold, and also disorders of fine motor skills (manipulation of objects).

Concerning pain disorders, the results clearly show that neuropathic pain is linked to cluster 3, and therefore to the severity of CIPN. Finally, concerning comorbidities, cluster 3 is mostly composed of patients whose CIPN has a significant impact on their HRQOL, and suffer from significant fatigue, insomnia and psychological distress (anxiety/depression). Our results are in line with the NCI-CTCAE classification of CIPN grades which indicate that a severity grade 3–4 incorporates a significant impact of CIPN on the patient’s daily life. This post-hoc study emphasized the intimate link between CIPN severity and psychological distress (Bao et al., 2016).

Based on our results, we propose to clinician some strategies to manage patients suffering from CIPN. Firstly, we encourage clinician to respect the guidelines of ESMO and ASCO, which inform that only duloxetine was effective to treat patients with chronic pain-related to CIPN (here cluster 3) (Jordan et al., 2020; Loprinzi et al., 2020; Derman and Davis, 2021). Nevertheless, it has been demonstrated that higher baseline emotional functioning predicted duloxetine response in a cohort of patients with oxaliplatin-induced CIPN (Smith et al., 2017). In our study, patients of cluster 3, who are suited to receive duloxetine, had the lowest scores of emotional functioning. Then, they would be more difficult to treat with duloxetine. This point underlines the expected difficulties to manage neuropathic pain in CIPN patients. Especially since recent studies in Japan (Hirayama et al., 2016, 2020), France (Selvy et al., 2020; 2021a; 2021b) and United States (Gewandter et al., 2020) have shown that oncologists do not prescribe duloxetine, or prescribe it very little, for the management of chronic pain related to CIPN. Secondly, we advise clinicians to consider particular attention to aggravating factors or factors related to the presence of severe CIPN, such as those listed above. This can be done with the help of specific questionnaires or even neurological explorations. But these explorations remain marginal and difficult to implement in a current practice and in the structures which do not have the equipment, an expert neurologist and/or the medical time necessary for this type of exploration. From our point of view, the association of patient-reported outcomes and clinician-reported outcomes, evaluating the whole biopsychosocial field of the patient, remains the most adequate strategy for the management of CIPN. At last, looking at the high levels of comorbidities (anxiety and depression) in the cluster 2 and cluster 3, a management of the psychological distress, in addition to the symptoms of CIPN, would be encourage for these patients.

In conclusion, our results support the importance of characterizing patients treated with oxaliplatin-based chemotherapy (and more broadly with neurotoxic chemotherapies), in order to identify characteristics aggravating or signaling the worsening of CIPN. These characteristics were high BMI, localization of neuropathic disorders in the lower limbs, fine motor impairment, presence of neuropathic pain, impairment of HRQOL, fatigue, insomnia, and psychological distress (anxiety/depression). Many of these features may be related to cancer alone and its management, making it difficult to diagnose CIPN and monitor the progression of its symptoms. Nevertheless, the characteristics of sensory, motor and vegetative disorders, including neuropathic pain, are signals that need to be finely characterized and assessed for each patient throughout chemotherapy in order to improve their management. This characterization and evaluation can be done using the various clinician reported outcomes (CROs) and/or patient reported outcomes (PROs) available to clinicians. Nevertheless, it is preferable to integrate the two evaluation systems because CROs underestimate the symptoms of CIPN and PROs may overestimate them (Alberti et al., 2014; Le-Rademacher et al., 2017; Jordan et al., 2020). Finally, it is important to add questionnaires on the psychological distress and HRQOL of the patients to these assessments that should also include a neurological assessment if possible (Jordan et al., 2020).

### Limitations of the Study

Some limitations may be discussed. The number of patients selected in cluster 3, and overall, may limit the significance of our secondary analyses. However, the very pronounced characteristics of cluster 3 allow identifying the highly neuropathic profile of these patients and suggest factors that worsen CIPN.

## Data Availability

The raw data supporting the conclusion of this article will be made available by the authors, without undue reservation.
