# Supplementary figures and images for: Colorectal Cancer Survivors Suffering From Sensory Chemotherapy-Induced Peripheral Neuropathy Are Not a Homogenous Group: Secondary Analysis of Patients’ Profiles With Oxaliplatin-Induced Peripheral Neuropathy
Source: Front Pharmacol. 2021 Nov 4;12:744085. doi: 10.3389/fphar.2021.744085 (PMC8599933; doi:10.3389/fphar.2021.744085)

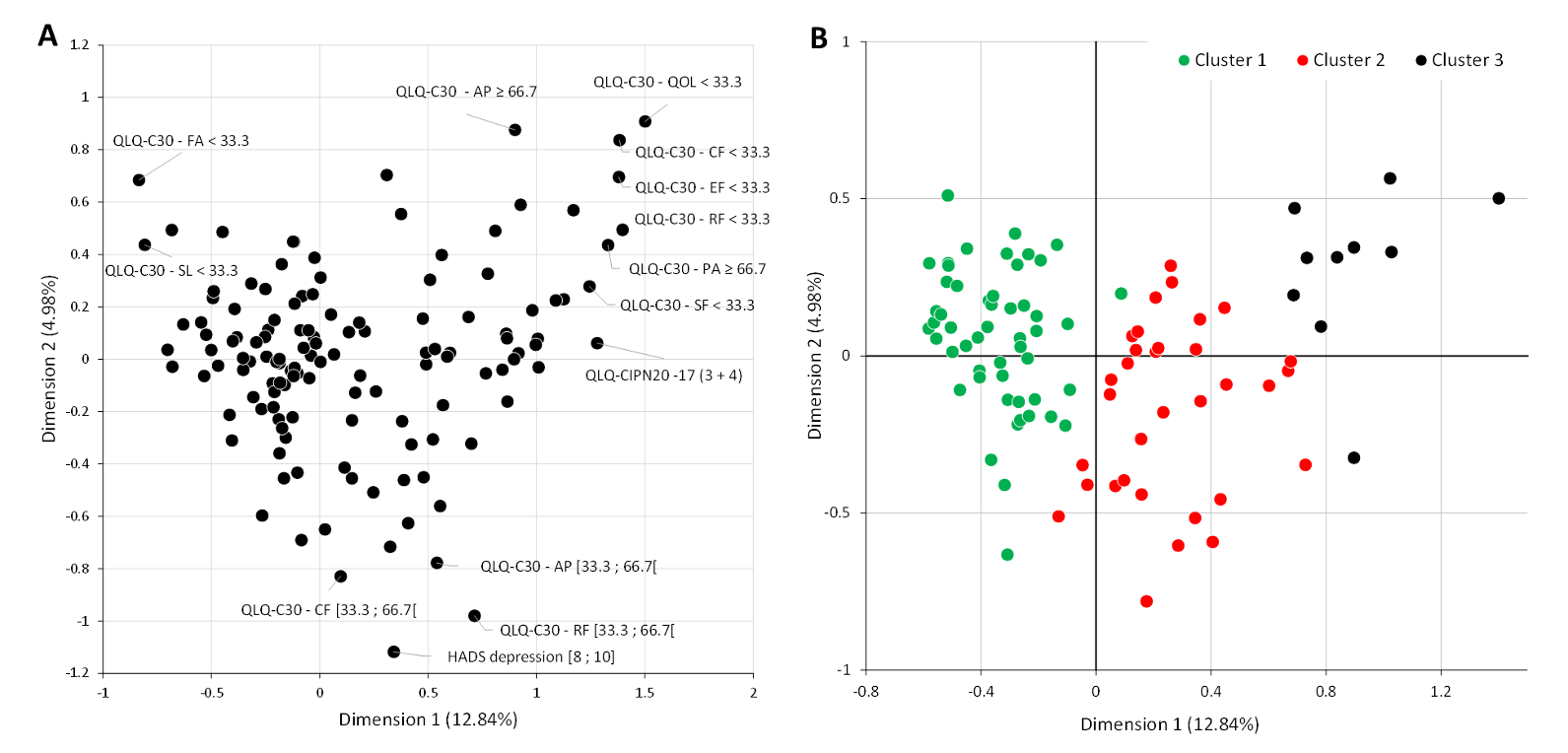

Supplement: Supplementary file 1 [file Image1.TIFF]
